# Supplementary material for: Development of a computer-aided design software for the quantitative evaluation of aesthetic damage
Source: PLoS One. 2019 Dec 18;14(12):e0226322. doi: 10.1371/journal.pone.0226322 (PMC6919621; doi:10.1371/journal.pone.0226322)
Supplement: S1 Code site — 1. (ZIP) [file pone.0226322.s001.zip › [EESC_jr][Projeto]Relatorio_07_21/vendors/select2/docs/_includes/examples/placeholders.html]

# Placeholders

A placeholder value can be defined and will be displayed until a
selection is made. Select2 uses the `placeholder` attribute
on multiple select boxes, which requires IE 10+. You can support it in
older versions with
the Placeholders.js polyfill.
